# Central Nervous System Double Relapse of Acute Promyelocytic Leukemia and Acute Myelomonocytic Leukemia

**DOI:** 10.1155/2019/4907352

**Published:** 2019-12-17

**Authors:** Laura M. Stanko, Vishnu Reddy, Fady M. Mikhail, Nikolaos Papadantonakis

**Affiliations:** ^1^Internal Medicine Program, University of Alabama at Birmingham, Birmingham, AL, USA; ^2^Division of Anatomic Pathology and Neuropathology, University of Alabama at Birmingham, Birmingham, AL, USA; ^3^Department of Genetics, University of Alabama at Birmingham, Birmingham, AL, USA; ^4^Division of Hematology/Oncology, University of Alabama at Birmingham, Birmingham, AL, USA

## Abstract

Relapse of acute promyelocytic leukemia (APL) and non-M3-acute myeloid leukemia in the central nervous system (CNS) are rare events. Here, we describe a case of simultaneous relapses of APL and acute myelomonocytic leukemia on the CNS of a patient after allogeneic bone marrow transplant. This extremely unusual case highlights the difficulties that CNS leukemia relapses pose in the post-transplant setting.

## 1. Introduction

Central nervous system (CNS) recurrence in adults with either acute promyelocytic leukemia (APL) or other subtypes of acute myeloid leukemia (AML) is uncommon, with previous studies reporting occurrences of 3–5% in APL and 1–2.5% in AML [1, 2]. These recurrences are typically associated with poor prognosis, and there is no consensus as to optimal treatment regimens [2, 3].

We present here a case of simultaneous, double relapse, of acute myelomonocytic leukemia (AMML) and APL in the CNS of a patient with concurrent molecular relapse of APL in the bone marrow (BM).

## 2. Case Presentation

### 2.1. Diagnosis of APL

The patient was 62 years old at the time of initial diagnosis of APL. She was hospitalized with bruising and malaise and found to have a white blood cell count (WBC) of 0.46 × 10^3^/cm^3^, hemoglobin of 8.6 gr/dl, and platelets of 13 × 10^3^/cm^3^. Based on the Sanz schema, she had intermediate risk APL [4]. Bone marrow (BM) biopsy was consistent with APL (Table 1). FISH analysis of peripheral blood demonstrated the presence of *PML/RARA* fusion in 95.5% of cells. The patient underwent induction chemotherapy with all-trans retinoic acid (ATRA) and idarubicin. A BM biopsy performed on day 35 was without morphological evidence of APL, but FISH analysis revealed 5.5% of interphase cells were still positive for the *PML/RARA* fusion. The karyotype was normal. The *PML/RARA* RT-PCR assay was positive (0.009).

The patient underwent consolidation with three cycles of ATRA and anthracycline (idarubicin and mitoxantrone were used). Repeat BM biopsy on day 50 was without morphological evidence of APL, and FISH analysis was negative.

BM biopsy on day 133 of APL diagnosis was consistent with molecular complete remission (mCR) (*PML/RARA* fusion RT-PCR product was undetectable). The patient then received two years of maintenance therapy with ATRA, 6-mercaptopurine, and methotrexate. A repeat BM biopsy at 37 months from diagnosis of APL was consistent with ongoing molecular CR (mCR).

### 2.2. Diagnosis of AMML

The patient presented 39 months after APL diagnosis with fatigue, dyspnea on exertion, and epistaxis. Laboratory workup revealed a hemoglobin 6.1 gr/dl, WBC 300 × 10^3^/cm^3^ with 86% blasts, and platelets 18 × 10^3^/cm^3^. The renal and liver function was within normal limits. The BM core biopsy revealed involvement by blasts. Karyotype was normal. FISH analysis for AML/MDS cytogenetic aberrations was normal. FISH analysis for *PML/RARA* translocation was negative. The *PML/RARA* fusion RT-PCR was also negative. Further analysis revealed that *CEBPA*, *FLT3-ITD*, and *C-Kit* mutations were not present; *FLT3-TKD* assay was positive. Flow cytometry identified two distinct populations (one showing CD34±, CD117+, CD13+, CD33+, CD15±, CD64±, CD14−, CD7−, CD2−, CD56−, and HLA-DRlo and a second population CD64+, CD14±, CD33+, and HLA-DRlo). Overall, the BM biopsy studies were consistent with AMML (Table 1).

Following a session of emergent leukapheresis, the patient underwent induction chemotherapy with FLAG regimen [fludarabine, high-dose cytarabine, and granulocyte colony-stimulating factor (G-CSF)]. Repeat BM biopsy one month after AMML diagnosis demonstrated CR. She then underwent consolidation with high-dose cytarabine (Ara-C), followed by haploidentical allogeneic bone marrow transplantation (alloHSCT) three months after diagnosis of AMML. The conditioning regimen included fludarabine, cyclophosphamide, and total body irradiation. She developed skin graft versus host disease (GVHD) controlled with topical steroids. She remained in CR for approximately 27 months following alloHSCT.

### 2.3. First Simultaneous Relapse of APL and AMML

**Figure 1:**
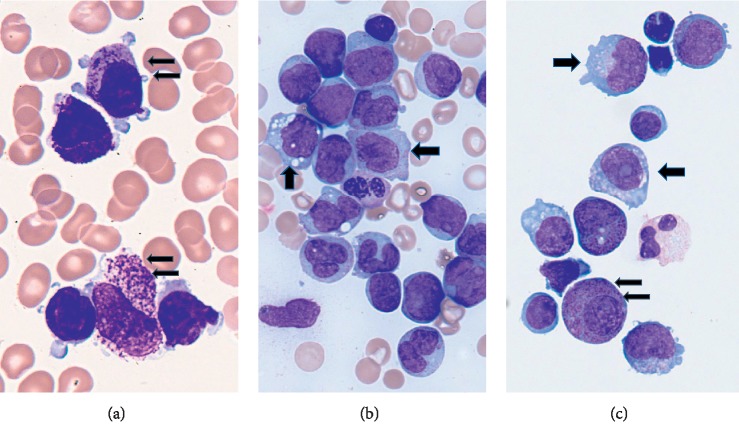
Initial APL blasts in the marrow (a). AMML occurrence in the bone marrow (b). CSF relapse showing myeloid/monoblasts (thick arrows) and APL blasts (small double arrows) (c).

**Figure 2:**
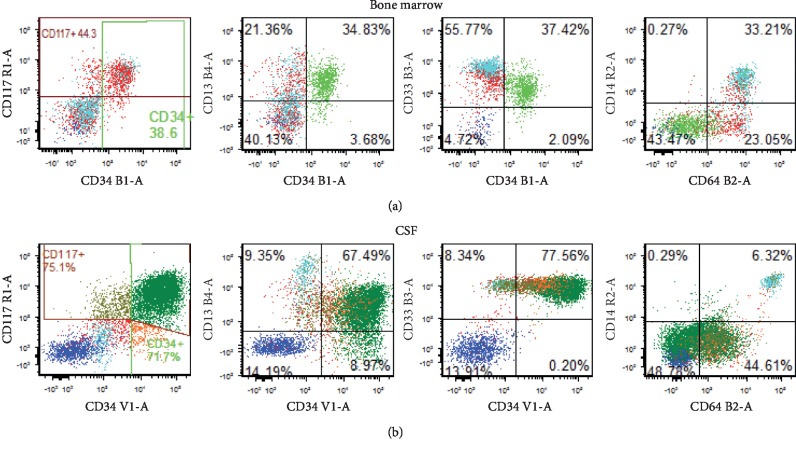
Flow cytometry: immunophenotypic similarity to AMML in the bone marrow at initial diagnosis (a) and CSF at relapse (b). Leukemic cells express of CD34, CD117, CD13, and CD64.

**Figure 3:**
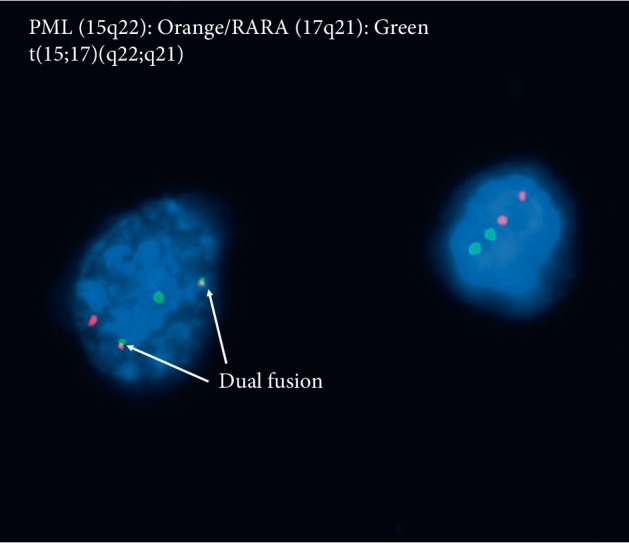
FISH analysis of CSF at double relapse.

The patient presented to the emergency department approximately 70 months after initial APL diagnosis and 30 months after AMML diagnosis with headache, nausea, and vomiting. CBC on presentation showed WBC 7.9 × 10^3^/cm^3^, hemoglobin 14.2 gr/dl, and platelets 146 × 10^3^/cm^3^. An MRI brain showed abnormal leptomeningeal enhancement in the posterior fossa as well as over both cerebral convexities with abnormal signal within the calvaria and upper cervical vertebra worrisome for leukemic infiltration of the marrow. Lumbar puncture was performed which revealed protein 217 mg/dl, glucose 73 mg/dl, and WBC 1,004 cm^3^. CSF studies were positive for the presence of both myelomonocytic and APL blasts (Figures 1 and 2; Table 2). The FISH analysis performed on CSF cells showed that cells harbored the *PML/RARA* translocation (Figure 3). BM biopsy did not reveal dysplasia or increased blasts by morphology or flow cytometry. The karyotype was normal. FISH analysis was negative for *PML/RARA* translocation. However, *PML/RARA* fusion RT-PCR was detected in the BM biopsy sample (with a value of 28.692); notably, the *PML/RARA* fusion RT-PCR was undetectable in a peripheral blood sample. The CT scan of the chest/abdomen/pelvis with contrast was without suspicious findings for extramedullary disease. Overall, the findings were compatible with CNS relapse of APL and AMML and molecular relapse of APL in the BM.

The patient received IT triple therapy (cytarabine, methotrexate, and hydrocortisone) and high-dose Ara-C while hospitalized (2.0 gr/m^2^ every 12 hours for a total of 8 doses). Repeat CSF studies six days after chemotherapy initiation showed a decrease in WBC to 48/cm^3^. Repeat MRI brain 14 days after presentation showed interval resolution of the leptomeningeal enhancement with persistent BM heterogenicity. A repeat BM biopsy, at count recovery, remained without morphological or immunophenotypic evidence of AMML or APL. The *PML/RARA* fusion RT-PCR was negative.

We then formulated a treatment plan based on the European guidelines for APL (2007) including ATO and ATRA. She received a course of ATRA (45 mg/m^2^ in two divided doses) and 28 doses of ATO (0.15 mg/kgr) in a 28-day cycle. Repeat BM biopsy after this course confirmed that the patient remained in mCR. We then proceeded to two weeks of ATO/ATRA administration that were repeated every four weeks. She completed six cycles, and BM biopsy confirmed ongoing mCR. The patient received ATRA for two weeks every three months for 2 cycles (5 months total).

### 2.4. Second Simultaneous Relapse of APL and AMML

Approximately 17 months after CNS relapse of APL and AMML and 87 months after initial APL diagnosis, the patient began experiencing headaches.

The CSF studies revealed WBC of 483 cm^3^ and protein was 91 mg/dl (Table 2). Flow cytometry and FISH analysis for *PML/RARA* translocation in the CSF supported second simultaneous relapse of AMML and APL (Table 2). A BM biopsy was without morphological or immunophenotypic evidence of APL or AMML. PML/RARA fusion was again detected (level was 12.327), but FISH analysis for PML/RARA translocation and the karyotype were normal.

The patient completed 6 IT triple therapy treatments with clearance of her CSF. The patient also concurrently began ATRA 45 mg/m^2^ in two divided doses and ATO (0.15 mg/kgr) for 28 doses. Repeat BM biopsy was without evidence of APL (mCR).

Despite the multiple treatments, our patient has not required hospitalizations after the high-dose cytarabine and did not experience significant toxicities.

## 3. Discussion

Recurrence of APL or non-M3-AML in the CNS is rare [5]. Here, we describe the simultaneous recurrence of both APL and AMML in the CNS of a patient. The rarity of non-M3-AML CNS involvement was highlighted in a study of 3,261 patients; 21 patients were identified at diagnosis and 34 at relapse [6]. In the study of Oshima et al. [7], CNS relapse in patients with leukemia that had underwent alloHSCT was described. They identified nine relapses in 533 patients with AML. In multivariate analysis, active disease at transplantation and history of CNS involvement were associated with CNS relapse.

In another report, outcomes of AML-isolated CNS recurrence of 34 patients, of which 18 had meningeal involvement, were described [3]. These patients underwent IT chemotherapy with methotrexate and dexamethasone plus cytarabine twice weekly until CR achieved; they were then given at least five additional monthly IT doses. Ultimately, 14 out of the 18 patients achieved CNS-CR but unfortunately, the median overall survival of the 34 patients was 6.64 months [3]. Cheng et al. also conducted a retrospective study of AML patients (excluding FAB M3), demonstrating 5.1% of the 395 patients developed CNS disease with only 1% (four patients) having an isolated CNS relapse [1]. This study showed three out of the four patients also developed subsequent BM relapses and died. The median overall survival was 8.5 months from relapse [1]. Taken together, the involvement of CNS by non-M3-AML is rare and is associated with poor outcomes.

APL CNS relapse has been reported in cohort studies and in case series [8–10]. Eleven patients out of the 739 enrolled in PETHEMA LPA96 and LPA99 trials had documented CNS APL relapse [11]. The median time of CNS relapse was 16 months, and authors reported leukocytosis at diagnosis and CNS hemorrhage as risk factors. Notably, from the seven patients with CNS involvement without morphological involvement of the bone marrow, 5 patients experienced relapse (CNS or BM). Few case reports have reported CNS relapse after alloHSCT [11, 12]. In our patient, the relapse of APL in the CNS was also accompanied by BM molecular relapse given the presence of *PML/RARA* fusion RT-PCR product. Notably, it has been previously reported that CNS relapse of APL can be accompanied by systemic relapse [13]. There are no consensus guidelines for the treatment of CNS APL with systemic relapse [14], and different strategies can be employed.

For example, in the report of Vega-Ruiz et al. [2], seven patients with recurrence of APL in the CNS were described. CNS relapse was typically accompanied by or preceded by systemic relapse. All seven patients were treated with intrathecal chemotherapy and systemic chemotherapy (six out of the seven patients died within four months of development relapse) [2].

Our patient was treated for both APL and M4-AML relapse with a treatment regimen that includes intrathecal chemotherapy, high-dose ARA-C, and consolidation and postconsolidation phase based on ATO and ATRA. Prolonged maintenance was initiated and was based on ATRA. Unfortunately, the patient experienced a second simultaneous relapse in CNS as well as molecular relapse in the bone marrow.

For patients with relapsed APL, autologous transplantation is recommended, but our patient relapsed after alloHSCT. Given her recent relapse, she will be evaluated for possible donor lymphocyte infusion.

It is intriguing that this patient developed two acute leukemias that relapsed simultaneously. The CNS may escape from the graft versus leukemia effect, but the optimal strategy to avoid relapse in CNS has not been identified.

## 4. Conclusion

This case describes a double recurrence of APL and M4-AML in the CNS of the patient after alloHSCT, accompanied by APL molecular relapse in the bone marrow. Our patient responded transiently to combined schema of IT chemotherapy and ATO/ATRA for more than a year. Our case highlights the difficulty that relapsed APL and CNS recurrence of leukemia can pose. In addition, it highlights that a combination of treatments may lead to remissions with meaningful duration and without significant toxicities.

## Figures and Tables

**Table 1:** Key studies at APL and AMML diagnosis.

BM studies	APL diagnosis	AMML diagnosis
Flow cytometry	CD34+, CD33+, CD117+, CD38+, and HLA-DR in majority of blasts.	Flow cytometry analysis of the peripheral blood revealed a population of immature myeloid and monocytic lineage cells accounting for 28% of the total cells with the following immunophenotypes: CD34±, CD117+, CD13+, CD33+, CD15 heterogeneous, CD64 heterogeneous, CD14−, CD7−, CD2−, CD56−, and HLA-DRlo. The monocytic lineage showed an increased population of immature cells (48% of cells CD64+, CD14±, CD33+, and HLA-DRlo, with heterogeneous expression of CD117, CD13, and CD15).

Morphological description	(i) Hypercellular marrow, >95% cellularity, and mostly replaced by leukemic infiltrates. Cytoplasmic granules were noticed in subsets of blasts. A small subset of blasts had cytoplasmic hypergranulation with rare Auer rods present. Scattered blasts also show bilobed nuclear morphology. Examination of the clot section and core biopsy showed hypercellular marrow (near 100%), replaced by sheets of immature cells. Subsets of the immature cells showed morphologic features of promyelocytes. No residual hematopoiesis was noted. (ii) These findings were consisted with APL, hypogranular type.	(i) Hypercellular (90%) marrow with blasts occupying in excess of 90% of the marrow space. The cellular elements were almost exclusively made up of blasts (>90%). No blasts with APL morphology were noted. Some of the blasts had round nucleus with fine chromatin and moderate cytoplasm (myeloid blasts) and others with more irregular and folded nuclei, fine chromatin, and abundant cytoplasm. Some blasts contained Auer rods. (ii) Overall, the BM biopsy was consistent with acute myeloid leukemia with myelomonocytic phenotype.

Cytogenetics	The t (15;17) was detected in 95.5% of analyzed cells.	Karyotype: 46, XY[20]. FISH analysis for *PML/RARA* translocation was normal.

**Table 2:** Key CBC and CSF studies during patient course.

	CSF WBC (cm^3^)	CSF RBC (cm^3^)	CSF protein (mg/dl)	Flow cytometry analysis	FISH analysis	WBC (×10^3^/cm^3^)	Hgb (gr/dl)	Platelets (×10^3^/cm^3^)
First APL/AMML double relapse	1,0004 (normal range: 0–5)	0 (normal range: 0-1)	217 (normal range: 18–53)	Flow cytometric analysis revealed a dysplastic population of myeloblasts comprising 19% of the total cells that are CD34+, CD117+, CD13+, CD33+, CD15−, CD64−, and CD14−	30% of the cells had the t (15;17)(q22;q21) translocation. Only 50 interphase cells were evaluated	7.9 (normal range: 4–11)	14.2 (normal range: 11.3–15.2)	146 (normal range: 150–440)
Second APL/AMML double relapse	483 (normal range: 0–5)	0 (normal range: 0-1)	91 (normal range: 18–53)	Wright-stained cytospin revealed numerous blasts. Many had abundant cytoplasm, and rare blasts contain cytoplasmic granules. No definite abnormal promyelocytes noted. Flow cytometric analysis revealed an aberrant population of myeloblasts comprising 72% of the total cells that are CD34+, CD117+, CD38−, CD13+, CD33+ (96.7), CD15−, CD64−, CD14−, CD7+, CD56 heterogenous, HLA-DRlo/+, and CD123+ (92.5%)	60% of the cells had the t (15;17)(q22;q21) resulting in *PML*/*RARA* fusion	7.42 (normal range: 4–11)	13.3 (normal range: 11.3–15.2)	227 (normal range: 150–440)

## References

[1] Cheng C. L., Li C. C., Hou H. A. (2015). Risk factors and clinical outcomes of acute myeloid leukaemia with central nervous system involvement in adults. *BMC Cancer*.

[2] Vega-Ruiz A., Faderl S., Estrov Z. (2009). Incidence of extramedullary disease in patients with acute promyelocytic leukemia: a single-institution experience. *International Journal of Hematology*.

[3] Zheng C., Liu X., Zhu W., Cai X., Wu J., Sun Z. (2014). Tailored central nervous system-directed treatment strategy for isolated CNS recurrence of adult acute myeloid leukemia. *Hematology*.

[4] Sanz M. A., Lo Coco F., Martín G. (2000). Definition of relapse risk and role of nonanthracycline drugs for consolidation in patients with acute promyelocytic leukemia: a joint study of the PETHEMA and GIMEMA cooperative groups. *Blood*.

[5] Albano F., Specchia G. (2011). Extramedullary disease in acute promyelocytic leukemia: two-in-one disease. *Mediterranean Journal of Hematology and Infectious Diseases*.

[6] Alakel N., Stölzel F., Mohr B. (2017). Symptomatic central nervous system involvement in adult patients with acute myeloid leukemia. *Cancer Management and Research*.

[7] Oshima K., Kanda Y., Yamashita T. (2008). Central nervous system relapse of leukemia after allogeneic hematopoietic stem cell transplantation. *Biology of Blood and Marrow Transplantation*.

[8] Evans G., Grimwade D., Prentice H. G., Simpson N. (1997). Central nervous system relapse in acute promyelocytic leukaemia in patients treated with all-trans retinoic acid. *British Journal of Haematology*.

[9] Burry L. D., Seki J. T. (2002). CNS relapses of acute promyelocytic leukemia after all-trans retinoic acid. *The Annals of Pharmacotherapy*.

[10] Seki S. H., Ryoo H. M., Cho H. S., Lee J. L., Lee K. H., Hyun M. S. (2004). Meningeal relapse in a patient with acute promyelocytic leukemia: a case report and review of the literature. *Journal of Korean Medical Science*.

[11] Montesinos P., Diaz-Mediavilla J., Deben G. (2009). Central nervous system involvement at first relapse in patients with acute promyelocytic leukemia treated with all-trans retinoic acid and anthracycline monochemotherapy without intrathecal prophylaxis. *Haematologica*.

[12] Classen C. F., Debatin K.-M., Friedrich W., Schulz A. S. (2003). Long-term remission of APL with a second allogeneic BMT after CNS relapse following HLA-identical allogeneic BMT. *Bone Marrow Transplantation*.

[13] Lee H. Y., Kim K. M., Kang M.-H., Kang J. H., Kang K.-M., Lee G.-W. (2006). Concurrent craniospinal radiotherapy and intrathecal chemotherapy in patient with acute promyelocytic leukemia second relapsed in central nervous system (CNS) following allogeneic stem cell transplantation. *Journal of Neuro-Oncology*.

[14] Tallman M. S., Altman J. K. (2009). How I treat acute promyelocytic leukemia. *Blood*.

